# Investigation of carbapenemases and aminoglycoside modifying enzymes of *Acinetobacter baumannii* isolates recovered from patients admitted to intensive care units in a tertiary-care hospital in Brazil

**DOI:** 10.1590/0037-8682-0094-2019

**Published:** 2019-12-20

**Authors:** Milena Polotto, Tiago Casella, Fernanda Modesto Tolentino, Mayra Mioto Mataruco, Naiady Konno Madela Porto, Mirella Fontana Batista Binhardi, Mara Corrêa Lelles Nogueira

**Affiliations:** 1 Instituto Adolfo Lutz, Centro de Laboratório Regional de São José do Rio Preto, São José do Rio Preto, SP, Brasil.; 2 Faculdade de Medicina de São José do Rio Preto, Centro de Investigação de Microrganismos, São José do Rio Preto, SP, Brasil.; 3 Universidade Federal de São Carlos, São Carlos, SP, Brasil.

**Keywords:** Acinetobacter baumannii, Carbapenemases, Aminoglycoside modifying enzymes

## Abstract

**INTRODUCTION:**

*Acinetobacter baumannii* are opportunistic bacteria, highly capable of acquiring antimicrobial resistance through the production of carbapenemases and aminoglycoside modifying enzymes (AMEs).

**METHODS:**

Carbapenemase and AME genes were investigated in *A. baumannii* recovered from inpatients of a Brazilian hospital.

**RESULTS:**

The key genes found were *bla*_OXA-51-like,_ the association ISAba1- *bla*_OXA-23-like_, and the AME genes *aph(3´)-VI, aac(6´)-Ib, aac(3)-Ia*, and *aph(3´)-Ia.* Different clusters spread through the institution wards.

**CONCLUSIONS:**

The dissemination of *bla*_OXA-23-like_ and AME-carrying *A. baumannii* through the hospital highlights the need for improved preventive measures to reduce the spread of infection.

*Acinetobacter baumannii*are opportunistic pathogens that cause nosocomial infections mainly in immunocompromised patients and those admitted to intensive care units (ICUs). This microorganism is highly capable of surviving and spreading through hospital environments and acquiring resistance to multiple classes of antimicrobials^1^. *A. baumannii* is considered to be one of the six particularly problematic pathogens in terms of antimicrobial availability issues arising from drug resistance, and its multidrug-resistant clones have spread globally^1^.

Carbapenem antibiotics have been widely used for treating *A. baumannii* infections; however, the emergence of resistant strains has been increasingly reported and is often associated with high mortality rates and a substantial increase in hospitalization costs^2^. Moreover, as carbapenem-resistant (CR) *A*.*baumannii* also presents resistance to other antimicrobial classes, alternative therapeutic approaches need to be explored^3^. The most commonly identified mechanism of carbapenem resistance among*A. baumannii*isolates is the production of oxacillinases (OXAs), which include OXA-23-like, OXA-24-like, OXA-58-like, and the intrinsic OXA-51-like enzymes^4^.

Aminoglycosides are a potential treatment option for CR *A. baumannii* infections^3^; however, several isolates are capable of producing aminoglycoside-modifying enzymes (AMEs), the most important aminoglycoside resistance mechanism in *A. baumannii* ^5^. Information about aminoglycoside resistance and the prevalence of AME-producing *A. baumannii* in Brazil is scarce, and few studies have been conducted to investigate these enzymes in this species.

Thus, the objectives of this study were to investigate the prevalence and diversity of carbapenemases and AME-encoding genes in CR *A*.*baumannii* isolates, and to evaluate the clonal relationships among them. 

The study was conducted from July to December 2012 at a 746-bed public tertiary-care teaching hospital in São José do Rio Preto (São Paulo State, Brazil). The hospital receives patients from more than 100 cities in this region and has 117 ICU beds.

One hundred non-duplicated CR *A. baumannii* isolates were obtained from different clinical specimens from patients admitted to 6 different hospital ICUs (Cardiology-ICU, Emergency-ICU, General private-ICU, General public-ICU, Pediatric-ICU, and Semi-intensive-ICU). Initially, identification of the *Acinetobacter baumannii-Acinetobacter calcoaceticus* complex was performed using the VITEK-2 Compact system (bioMérieux, Marcy-l'Étoile, France). For species confirmation, a duplex-polymerase chain reaction (PCR) was used to detect the specific intergenic region present in *A. baumannii*. Primers (5'-CATTATCACGGTAATTAGTG and 5'-AGAGCACTGTGCACTTAAG) were used to amplify a specific internal 208 bp fragment from the Intergenic Specific region of*A. baumannii*, and a highly conserved 425 bp region of the*recA* gene was used as a control^6^ (5'-CCTGAATCTTCTGGTAAAAC and 5'-GTTTCTGGGCTGCCAAACATTAC).

Antimicrobial susceptibility testing was performed by microdilution using the VITEK-2 Compact system for the following antimicrobials: ceftazidime, cefepime, meropenem, gentamicin, ciprofloxacin, and tigecycline. The disc-diffusion method was used to test imipenem, piperacillin/tazobactam, and amikacin susceptibility, and the broth microdilution method, Policimbac (Probac, São Paulo, Brazil), was used to test polymyxin B susceptibility. The results were analyzed according to the Clinical and Laboratory Standards Institute (CLSI) breakpoints (2014).

For DNA extraction, colonies of a recent pure culture (up to 24 hours) grown on MacConkey Agar plates were suspended in 500 μl of sterile water in a 1.5 ml tube, homogenized, and incubated at 95-99°C for 10 minutes. Next, this mixture was placed on ice for 3-5 minutes centrifuged at 16.1 rcf for 3 minutes; the supernatant was collected for PCR analysis. The carbapenemase genes *bla*_OXA-51-like_, *bla*_OXA-23-like_, *bla*_OXA-58-like_, *bla*_OXA-24-like_, *bla*_OXA-143-like_, *bla*_OXA-48-like_, *bla*_IMP_, *bla*_VIM_, *bla*_SPM_, and *bla*_KPC_, and the insertion sequence IS*Aba1* upstream of *bla*_OXA-23-like_, were all screened and sequenced using a 3130 Genetic Analyzer (Thermo Fisher Scientific). Three multiplex-PCRs were performed, as described previously^5^, to investigate the following AME genes: one triplex-PCR intended to amplify *aac(3)-Ia*, *aac(3)-IIa*, and *aac(6’)-Ih*, another triplex-PCR to amplify *aph(3’)-VI*, *ant(2”)-Ia*, and the internal control *rrn* gene, and a duplex-PCR to amplify *aph(3’)-Ia* and *aac(6’)-Ib*. All the AME genes detected were confirmed by sequencing. Conventional single PCRs, as described previously^7^, were used to screen the 16S rRNA methyltransferase genes *armA* and *rmtB*. Molecular relationships between the isolates carrying AME genes were evaluated by repetitive element sequence-based PCR (REP-PCR). Patterns were analyzed with BioNumerics® software version 6.1 (Applied Maths, Saint-Martens-Latem, Belgium), using the Dice band-based similarity coefficient and the unweighted pair group method with arithmetic mean as clustering methods. Isolates were assigned to the same cluster if their similarity coefficient was ≥ 90%. 

All *A. baumannii* isolates displayed a multidrug-resistant phenotype; in addition to carbapenems, isolates showed resistance to piperacillin/tazobactam, cefotaxime, ceftazidime, cefepime, and ciprofloxacin. The resistance rates to amikacin, gentamicin, tigecycline, and polymyxin B were 76%, 32%, 12%, and 2%, respectively. Bacterial isolates originated from tracheal aspirate (52%), catheter tips (22%), urine (8%), blood (5%), biopsy (4%), secretions (3%), pleural fluid (2%), and other clinical specimens (sputum, cerebrospinal fluid, bronchoalveolar lavage, and bone marrow;1% each). The isolates recovered from respiratory tract samples corresponded to 54% of collected CR *A. baumannii*. This species is an important agent related to mechanical ventilator-associated pneumonia (VAP), contracted from colonized patients, environmental surfaces, and other unknown sources^8^. In the present study, the respiratory tract was the most important source of CR *A. baumannii*, which is in accordance with several other countries^9^. The second greatest source of bacteria was catheter tips, which could be indicative of bloodstream infections; or contamination, as previous study reportedy^10^

All the CR *A. baumannii* were positive for *bla*_OXA-51-like_ and *bla*_OXA-23-like_ genes, and the detection of *bla*_OXA-51-like_ confirmed the species identity in all isolates^11^. High prevalence and dissemination of *bla*_OXA-23_ in *A. baumannii* have been reported worldwide, including Brazil^11^ ^,^ ^12^. Moreover, all isolates harbored the IS*AbaI* upstream of *bla*_OXA-23-like_ gene, which might be the cause of the observed carbapenem resistance^13^.

Among the AME-encoding genes, the most frequently detected was *aph(3´)-VI*, present in 55% of the isolates, followed by *aac(6´)-Ib*, detected in 47%, *aac(3)-Ia* in 27%, and *aph(3´)-Ia* in 22% of the isolates. All the other investigated carbapenemase and AME genes were not detected. Fifty percent of the isolates presented one AME gene, 26.7% presented two, and 24.4% presented three AME genes (Table 1). 

**TABLE 1:** Frequency of aminoglycoside modifying enzymes (AMEs) genotypes, substrate specificity, and aminoglycosides susceptibility phenotypes of the carbapenem resistant *A. baumannii*.

Genotype	Number of Isolates (%)	AME'ssubstrate	Resistance Phenotype (%)	
			Amikacin	Gentamicin
*aph(3')-VI*	36 (41.8)	AK	35/36 (97.2)	0
*aac(6')-Ib + aac(3)-Ia + aph(3')-Ia*	21 (24.4)	CN+AK	13/21 (61.2)	18/21 (85.7)
*aph(3')-VI + aac(6')-Ib*	17 (19.7)	AK	17/17 (100)	0
*aac(6')-Ib*	5 (5.8)	AK	5/5 (100)	0
*aac(6')-Ib + aac(3)-Ia*	3 (3.5)	AK+CN	1/3 (33.3)	3/3 (100)
*aac(3)-Ia*	2 (2.3)	CN	0	1/2 (50.0)
*aph(3')-VI + aac(3)-Ia*	2 (2.3)	AK+CN	2/2 (100)	2/2 (100)
*aac(6')-Ib + aph(3´)-Ia*	1 (1.1)	AK	1/1 (100)	0

AK: amikacin; CN: gentamicin.

The production of AMEs is the most important aminoglycoside resistance mechanism in *A. baumannii* ^3^. In this study, four AME genes were found, and two of them, *aph(3´)-VI* and *aac(6´)-Ib,* are known to be responsible for amikacin resitance^14^.

The majority of the isolates (97.2%) that harbored the *aph(3´)-VI* gene displayed resistance to amikacin (Table 1); the high frequency of amikacin inactivation by this enzyme has been previously described^14^. Among the 28 isolates that harbored the gentamicin resistance gene *aac(3)-Ia*, 85% were resistant to gentamicin, suggesting the production of the AAC(3)-Ia enzyme in these isolates. The high prevalence and coexistence of these AME genes circulating in the hospital environment is problematic as they limit the effectiveness of aminoglycoside usage as antimicrobials. Furthermore, these genes are highly mobile and are often transferred on plasmids, integrons, and transposons along with other resistance genes, such as *bla*_OXA-23-like_, enabling the spread of several resistance genes simultaneously^15^.

The molecular relationship between the 86 CR *A. baumannii* carrying AME genes was first elucidated by visual analysis of the dendrogram, in which the isolates were separated into 5 clusters (A-E); these were then divided into 19 sub-clusters named A1-A3, B1-B3, C1-C5, D1-D6, E1, and E2, according to similarity coefficients ≥ 90% [Supplementary data (Figure 1)]. Eight clusters (A2, A3, B2, B3, C3, C4, C5, and D5) were considered sporadic given their duration in days and the small number of isolates [Supplementary data (Figure 1)]. Thirty four percent of the *A. baumannii* isolates belonged to cluster B1, distributed across all wards over the duration of the study, indicating an endemic cluster. The inter-ward transmission and clonal spread of the *A. baumannii* isolates was evidenced in some clusters like A1 (isolates Ac18, Ac47, Ac57, and Ac59), B1 (isolates Ac08, Ac09, Ac11, Ac14, and Ac17), D1 (isolates Ac04, Ac05, and Ac58), and D6 (isolates Ac40 and Ac41), in which the isolates presented 100% similarity, and the same AME genes and aminoglycoside susceptibility profiles were observed [Supplementary data (Figure 1)]. 

Interestingly, some CR *A. baumannii* isolates presented different genotypes and susceptibility regarding aminoglycosides resistance, but exhibited 100% genotypic similarity to each other according to REP-PCR-typing, as observed in clusters A1 (Ac21 and Ac134), B1 (Ac30 and Ac54), C2 (Ac97, Ac100, and Ac139), and D4 (Ac50, Ac51, and Ac118) [Supplementary data (Figure 1)]. These discrepancies could be explained by the constant mobilization of AME genes, as they are often located in plasmids and class 1 integrons, suggesting that horizontal gene transfer plays an important role in the dissemination of aminoglycoside resistance in *A. baumannii* ^*15*^.

It is also relevant to note that the dendrogram presents two characteristic profiles. The first one, represented by the groups A, B, C, and E, that presents a similar and almost exclusive genotype of AME genes (*aph(3´)-VI* and occasionally *aac(6´)-Ib*), with the majority of these members presenting resistance only to amikacin ([Supplementary data (Figure 1)]). The second profile, represented by group D, the most diverse group, presents a general common genotype of AME genes among its members (*aac(6´)-Ib*, *aac(3)-Ia,* and *aph(3´)-Ia*), and resistance to gentamicin and intermediate resistance to amikacin [Supplementary data (Figure 1)]. 

Another interesting finding is related to group E, which presented lower similarity (46.7%) to groups A, B, C, and D. Its first isolate, Ac133 (cluster E1), was recovered on November 29 from the Emergency-ICU, where new severe inpatients are admitted; three days later, two isolates of the same E1 cluster (Ac130 and Ac138) were recovered from the General-ICU [Supplementary data (Figure 1)]. This may have occurred due to the introduction of a new cluster into the hospital, since their isolates were recovered only in the final period, and a rapid dissemination may have happened between the two ICUs. 

In conclusion, we consider that a clonal dissemination of multidrug-resistant *A. baumannii* occurred within the institution. This finding emphasizes the need for improved infection control, such as isolation of colonized or infected patients and environmental disinfection interventions, to prevent further dissemination of *A. baumannii* in the institution.
