# Supplementary material for: Investigation of carbapenemases and aminoglycoside modifying enzymes of Acinetobacter baumannii isolates recovered from patients admitted to intensive care units in a tertiary-care hospital in Brazil
Source: Rev Soc Bras Med Trop. 2019 Dec 20;53:e20190094. doi: 10.1590/0037-8682-0094-2019 (PMC7083385; doi:10.1590/0037-8682-0094-2019)
Supplement: Supplementary file 1 [file 1678-9849-rsbmt-53-e20190094-suppl1.pdf]

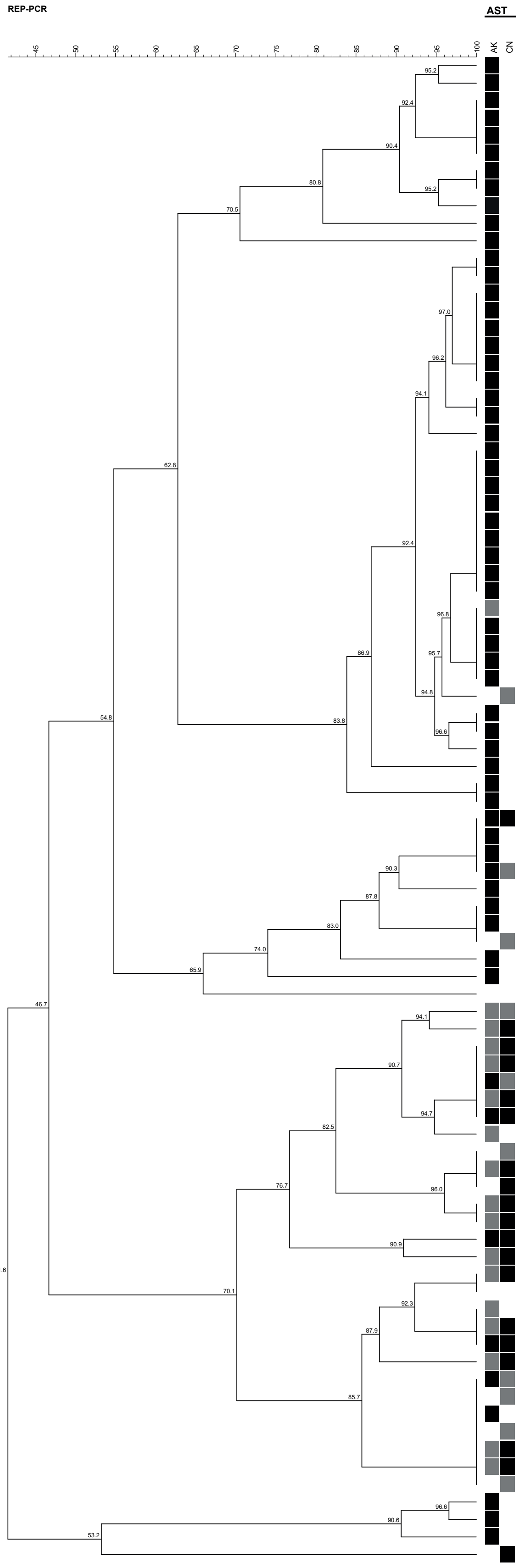

Cluster	Isolate	Source	Isolation Date	AMEs Genotype	Ward
A1	Ac 29	Wound	Aug/2012	aph(3')-VI, aac(6')-Ib	Emergency
A1	Ac 65	Tracheal aspirate	Sep/2012	aph(3')-VI	Semi-intensive
A1	Ac 18	Urine	Aug/2012	aph(3')-VI	Emergency
A1	Ac 47	Tracheal aspirate	Sep/2012	aph(3')-VI	Emergency
A1	Ac 57	Catheter tip	Sep/2012	aph(3')-VI	Semi-intensive
A1	Ac 59	Tracheal aspirate	Sep/2012	aph(3')-VI	Semi-intensive
A1	Ac 21	Tracheal aspirate	Aug/2012	aph(3')-VI	Emergency
A1	Ac 134	Catheter tip	Nov/2012	aph(3')-VI, aac(6')-Ib	General public
A1	Ac 69	Catheter tip	Oct/2012	aph(3')-VI	Emergency
A2	Ac 106	Blood	Oct/2012	aph(3')-VI, aac(6')-Ib	General public
A3	Ac 150	Urine	Dec/2012	aph(3')-VI, aac(6')-Ib	General public
B1	Ac 30	Tracheal aspirate	Aug/2012	aph(3')-VI, aac(6')-Ib	General public
B1	Ac 54	Blood	Sep/2012	aph(3')-VI	General public
B1	Ac 08	Tracheal aspirate	Aug/2012	aph(3')-VI	General public
B1	Ac 09	Catheter tip	Aug/2012	aph(3')-VI	General public
B1	Ac 11	Tracheal aspirate	Aug/2012	aph(3')-VI	Semi-intensive
B1	Ac 14	Tracheal aspirate	Aug/2012	aph(3')-VI	Emergency
B1	Ac 17	Tracheal aspirate	Aug/2012	aph(3')-VI	Emergency
B1	Ac 36	Sputum	Aug/2012	aph(3')-VI, aac(6')-Ib	General public
B1	Ac 01	Pleural fluid	Jul/2012	aph(3')-VI	Pediatric
B1	Ac 02	Tracheal aspirate	Jul/2012	aph(3')-VI	General priv.
B1	Ac 39	Tracheal aspirate	Aug/2012	aph(3')-VI, aac(6')-Ib	Emergency
B1	Ac 63	Tracheal aspirate	Sep/2012	aph(3')-VI	General public
B1	Ac 73	Secretion	Sep/2012	aph(3')-VI	Cardiological
B1	Ac 93	Tracheal aspirate	Oct/2012	aph(3')-VI, aac(6')-Ib	General priv.
B1	Ac 96	Tracheal aspirate	Oct/2012	aph(3')-VI	General public
B1	Ac 109	Tracheal aspirate	Oct/2012	aph(3')-VI	Emergency
B1	Ac 112	Tracheal aspirate	Oct/2012	aph(3')-VI	Cardiological
B1	Ac 120	Catheter tip	Nov/2012	aph(3')-VI, aac(6')-Ib	General priv.
B1	Ac 123	Tracheal aspirate	Nov/2012	aph(3')-VI, aac(6')-Ib	General public
B1	Ac 146	Tracheal aspirate	Dec/2012	aph(3')-VI	General priv.
B1	Ac 03	Bone	Jul/2012	aph(3')-VI	Emergency
B1	Ac 52	Catheter tip	Sep/2012	aph(3')-VI	General public
B1	Ac 06	Tracheal aspirate	Jul/2012	aph(3')-VI	Semi-intensive
B1	Ac 44	Catheter tip	Sep/2012	aph(3')-VI, aac(6')-Ib	General public
B1	Ac 48	Catheter tip	Sep/2012	aph(3')-VI, aac(6')-Ib	General public
B1	Ac 119	Tracheal aspirate	Nov/2012	aac(3)-Ia, aph(3')-Ia	General public
B1	Ac 95	Tracheal aspirate	Oct/2012	aph(3')-VI	Semi-intensive
B1	Ac 105	Tracheal aspirate	Oct/2012	aph(3')-VI	General public
B1	Ac 53	Tracheal aspirate	Sep/2012	aph(3')-VI	Cardiological
B2	Ac 82	Biopsy	Oct/2012	aph(3')-VI, aac(6')-Ib	General public
B3	Ac 147	Secretion	Dec/2012	aph(3')-VI	Semi-intensive
B3	Ac 148	Tracheal aspirate	Dec/2012	aph(3')-VI	General public
C1	Ac 12	Urine	Aug/2012	aph(3')-VI	Semi-intensive
C1	Ac 26	Tracheal aspirate	Aug/2012	aac(3)-Ia, aph(3')-Ia	Emergency
C1	Ac 61	Tracheal aspirate	Sep/2012	aph(3')-VI	General public
C1	Ac 62	Catheter tip	Sep/2012	aph(3')-VI	General public
C1	Ac 77	Tracheal aspirate	Oct/2012	aph(3')-VI	General public
C2	Ac 97	Tracheal aspirate	Oct/2012	aph(3')-VI, aac(6')-Ib	Cardiological
C2	Ac 100	Tracheal aspirate	Oct/2012	aph(3')-VI, aac(6')-Ib	Cardiological
C2	Ac 139	Catheter tip	Nov/2012	aac(3)-Ia	Semi-intensive
C3	Ac 137	Blood	Nov/2012	aph(3')-VI	Cardiological
C4	Ac 128	Tracheal aspirate	Nov/2012	aph(3')-VI	Cardiological
C5	Ac 115	Tracheal aspirate	Oct/2012	aac(3)-Ia	Emergency
D1	Ac 49	Tracheal aspirate	Sep/2012	aac(6')-Ib; aac(3)-Ia; aph(3')-Ia	General public
D1	Ac 114	Tracheal aspirate	Oct/2012	aac(6')-Ib; aac(3)-Ia; aph(3')-Ia	Emergency
D1	Ac 04	Brochoalveolar la.	Aug/2012	aac(6')-Ib; aac(3)-Ia; aph(3')-Ia	Emergency
D1	Ac 05	Tracheal aspirate	Aug/2012	aac(6')-Ib; aac(3)-Ia; aph(3')-Ia	General public
D1	Ac 45	Blood	Sep/2012	aac(6')-Ib	Emergency
D1	Ac 58	Catheter tip	Sep/2012	aac(6')-Ib; aac(3)-Ia; aph(3')-Ia	Emergency
D1	Ac 149	Urnie	Dec/2012	aac(6')-Ib; aac(3)-Ia; aph(3')-Ia	Emergency
D1	Ac 101	Bone marrow	Nov/2012	aac(6')-Ib	Semi-intensive
D2	Ac 78	Cerebrospinal fluid	Oct/2012	aac(6')-Ib; aac(3)-Ia; aph(3')-Ia	General public
D2	Ac 83	Tracheal aspirate	Oct/2012	aac(6')-Ib; aac(3)-Ia; aph(3')-Ia	General public
D2	Ac 86	Tracheal aspirate	Oct/2012	aac(6')-Ib; aac(3)-Ia; aph(3')-Ia	General priv.
D2	Ac 71	Urine	Sep/2012	aac(6')-Ib, aph(3')-Ia	Cardiological
D2	Ac 72	Tracheal aspirate	Sep/2012	aac(3)-Ia; aac(6')-Ib	Emergency
D3	Ac 81	Catheter tip	Oct/2012	aac(6')-Ib	General public
D3	Ac 127	Tracheal aspirate	Nov/2012	aac(6')-Ib; aac(3)-Ia; aph(3')-Ia	General public
D4	Ac 64	Tracheal aspirate	Sep/2012	aac(6')-Ib; aac(3)-Ia; aph(3')-Ia	Semi-intensive
D4	Ac 70	Tracheal aspirate	Sep/2012	aac(6')-Ib; aac(3)-Ia; aph(3')-Ia	Emergency
D4	Ac 50	Catheter tip	Sep/2012	aac(6')-Ib; aac(3)-Ia; aph(3')-Ia	General public
D4	Ac 51	Catheter tip	Sep/2012	aac(6')-Ib	General priv.
D4	Ac 118	Urine	Nov/2012	aph(3')-VI, aac(6')-Ib	Semi-intensive
D5	Ac 116	Tracheal aspirate	Nov/2012	aac(6')-Ib	Semi-intensive
D6	Ac 13	Tracheal aspirate	Aug/2012	aac(6')-Ib; aac(3)-Ia; aph(3')-Ia	General public
D6	Ac 16	Tracheal aspirate	Aug/2012	aac(6')-Ib; aac(3)-Ia; aph(3')-Ia	General public
D6	Ac 25	Tracheal aspirate	Aug/2012	aac(6')-Ib; aac(3)-Ia; aph(3')-Ia	Semi-intensive
D6	Ac 31	Catheter tip	Aug/2012	aac(6')-Ib; aac(3)-Ia; aph(3')-Ia	General public
D6	Ac 40	Pleural fluid	Aug/2012	aac(6')-Ib; aac(3)-Ia; aph(3')-Ia	General public
D6	Ac 41	Tracheal aspirate	Sep/2012	aac(6')-Ib; aac(3)-Ia; aph(3')-Ia	Emergency
D6	Ac 43	Tracheal aspirate	Sep/2012	aac(3)-Ia; aac(6')-Ib	Emergency
E1	Ac 130	Catheter tip	Dec/2012	aph(3')-VI, aac(6')-Ib	General public
E1	Ac 138	Tracheal aspirate	Dec/2012	aph(3')-VI, aac(6')-Ib	General public
E1	Ac 133	Tracheal aspirate	Nov/2012	aph(3')-VI	Emergency
E2	Ac 145	Catheter tip	Dec/2012	aph(3')-VI, aac(6')-Ib	Emergency

FIGURE 1: REP-PCR patterns of 86 CR *A. baumannii* carrying AMEs, and their respective susceptibility phenotypes to aminoglycosides (AS), source of isolation, and ICU. Squares represent resistance (black), intermediate susceptibility (gray), and sensitivity (white) to aminoglycosides.

OPEN ACCESS
https://creativecommons.org/licenses/by/4.0/
